# Correction: The solvation structure, transport properties and reduction behavior of carbonate-based electrolytes of lithium-ion batteries

**DOI:** 10.1039/d2sc90129c

**Published:** 2022-07-04

**Authors:** Tingzheng Hou, Kara D. Fong, Jingyang Wang, Kristin A. Persson

**Affiliations:** Department of Materials Science and Engineering, University of California Berkeley 210 Hearst Mining Building Berkeley California 94720 USA; Energy Technologies Area, Lawrence Berkeley National Laboratory Berkeley California 94720 USA; Department of Chemical and Biomolecular Engineering, University of California Berkeley CA 94720 USA; The Molecular Foundry, Lawrence Berkeley National Laboratory Berkeley California 94720 USA; Materials Sciences Division, Lawrence Berkeley National Laboratory Berkeley California 94720 USA kapersson@lbl.gov

## Abstract

Correction for ‘The solvation structure, transport properties and reduction behavior of carbonate-based electrolytes of lithium-ion batteries' by Tingzheng Hou *et al.*, *Chem. Sci.*, 2021, **12**, 14740–14751, https://doi.org/10.1039/D1SC04265C.

The original version of this manuscript contained typographical errors in the Conclusions. The anion–solvent exchange mechanism should be referred to as “exit-entry” type, not “entry-exit” type.

The sentence “We also reveal an anion–solvent exchange mechanism as “entry-exit” type, providing a dynamic perspective of ion transport in electrolytes” should therefore be “We also reveal an anion–solvent exchange mechanism as “exit-entry” type, providing a dynamic perspective of ion transport in electrolytes.”

The Royal Society of Chemistry apologises for these errors and any consequent inconvenience to authors and readers.

## Supplementary Material

